# Tobacco Use Changes and Perceived Health Risks among Current Tobacco Users during the COVID-19 Pandemic

**DOI:** 10.3390/ijerph18041795

**Published:** 2021-02-12

**Authors:** Jessica M. Yingst, Nicolle M. Krebs, Candace R. Bordner, Andrea L. Hobkirk, Sophia I. Allen, Jonathan Foulds

**Affiliations:** 1 Center for Research on Tobacco and Health, Department of Public Health Sciences, Penn State University College of Medicine, Hershey, PA 17033, USA; nkrebs@pennstatehealth.psu.edu (N.M.K.); cbordner@pennstatehealth.psu.edu (C.R.B.); ahobkirk@pennstatehealth.psu.edu (A.L.H.); sallen3@phs.psu.edu (S.I.A.); jfoulds@psu.edu (J.F.); 2 Center for Research on Tobacco and Health, Department of Psychiatry and Behavioral Health, Penn State University College of Medicine, Hershey, PA 17033, USA

**Keywords:** tobacco use, cigarette smoking, e-cigarettes, COVID-19, pandemic, quit attempts, cessation, stress

## Abstract

COVID-19 has become a global pandemic, with over 81 million cases worldwide. To assess changes in tobacco use as a result of the pandemic, we surveyed a convenience sample of current tobacco users between April and June 2020. The sample was taken from a tobacco user research registry (*n* = 3396) from the Penn State College of Medicine in Hershey, Pennsylvania, USA. Participants who responded to the survey and were eligible for this study (*n* = 291) were 25.6% male, 93% white, and had a mean age of 47.3 (SD = 11.6) years. There were no reports of participants testing positive for COVID-19, but 21.7% reported experiencing symptoms associated with the virus. Most participants (67%) believed that their risk of contracting COVID-19 was the same as non-tobacco users, but 57.7% believed that their risk of serious complications, if infected, was greater compared to non-tobacco users. A total of 28% reported increasing their cigarette use during the pandemic. The most common reasons for increased use were increased stress, more time at home, and boredom while quarantined. Nearly 15% reported decreasing their tobacco use. The most common reasons for reduced use were health concerns and more time around non-smokers (including children). A total of 71 (24.5%) users reported making a quit attempt. Characterizing these pandemic-related changes in tobacco use may be important to understanding the full scope of subsequent health outcomes resulting from the pandemic. Tobacco cessation resources should be tailored to allow for safe, appropriate access for those interested in quitting.

## 1. Introduction

In December 2019, the first cases of a novel coronavirus disease (COVID-19) were reported in Wuhan, Hubei province, China [1]. The disease quickly spread and has become a pandemic, with over 81 million cases and 1.7 million deaths worldwide as of 28 December 2020 [2]. COVID-19 is mainly a disease of the respiratory tract characterized by a severe acute respiratory syndrome (SARS-CoV-2) [1]. Cigarette smoking affects nearly every organ in the body, compromises the immune system, and is associated with increased risk for pulmonary infections [3]. Smokers admitted to the hospital with SARS-CoV-2 are at a higher risk for complications and death from the respiratory COVID-19 virus [4], although some evidence suggests that nicotine may have a protective effect against initial COVID-19 infection [5,6,7]. Conditions such as lung disease, heart disease, diabetes, and obesity are all associated with tobacco use, and are linked with higher rates of hospitalization and mortality from COVID-19 infection [8,9]. Thus, tobacco use remains a serious concern during the pandemic.

The pandemic has abruptly changed the way people live by way of social distancing, abiding by stay-at-home orders, quarantining, and by the shutting down of businesses and social gatherings. These circumstances provoked by the pandemic have resulted in increased stress and anxiety over an uncertain future [10,11]. Given that tobacco use is influenced by negative affective states such as stress [12,13,14], environmental conditions that facilitate or restrict cigarette access and use [15], and harm perceptions [16,17], it is important to understand tobacco use changes in response to the pandemic. For example, the threat of COVID-19 could result in negative consequences such as increased tobacco use, or users could have a renewed interest in quitting as a proactive step to improve their health. Historically, other national disasters have resulted in relapse in former smokers or increase in smoking among current smokers [18,19,20,21]. Additionally, evidence suggests an increased interest in cessation assistance among smokers after experiencing natural disasters [21].

As most studies related to tobacco use during the COVID-19 pandemic have mainly focused on the epidemiological evidence for estimates of infection and disease, research on tobacco use changes due to the pandemic in the United States (U.S.) is currently limited. An analysis of mostly non-daily dual cigarette and e-cigarette users found that approximately 30% increased their product use while others reduced use [22]. Among college students, campus closures related to COVID-19 were associated with reduced tobacco use frequency (i.e., days) but not actual quantity [23]. Finally, a multi-country study that included the U.S. found that there was a stronger desire to quit smoking or vaping if someone had a direct experience with COVID-19, such as someone in their household testing positive [24].

Our study aimed to build on this evidence detailing the behavior changes of tobacco users in central Pennsylvania during the onset of the pandemic. We evaluated the behavior changes and perceptions among current tobacco users during the COVID-19 pandemic using an online survey. This included evaluating the frequency of use, reasons for changes in use, and attempts to quit. Unique to this study, we evaluated qualitative data to further elucidate reasons for changes in use. It is important to characterize these changes as they could identify opportunities or barriers to tobacco cessation during these times of economic, psychological, and social hardships. We also evaluated perceptions of COVID-19 health risks, as they could be related to tobacco use behavior changes.

## 2. Materials and Methods

### 2.1. Participants

Survey respondents were a convenience sample of members in a tobacco user research registry who agreed to be contacted for future tobacco-related research at the Penn State College of Medicine. Participants were recruited from the central Pennsylvania area using postal flyers, social media, bus advertisements, and word of mouth. Participants were entered into the registry from August 2015 to April 2020. Email invitations with a unique survey link were sent to the participants in this registry on 23 April 2020. This was approximately one month after the Pennsylvania governor ordered the temporary closure of all non-life-sustaining businesses and issued the first stay-at-home orders. Two subsequent reminder emails were sent weekly to those who did not respond to the initial invitation. 

Emails were sent to a total of 3396 valid email addresses and 406 (12.0%) participants completed the survey. Participants included in the analysis were those who reported either current cigarette or e-cigarette use pre-COVID. In addition, all participants included were at least 21 years of age and could read and write in English. Participants were removed from analysis for the following reasons: pre-COVID tobacco use was not reported (*n* = 79), reported no current tobacco use pre-COVID (*n* = 31), not a cigarette or e-cigarette user pre-COVID (*n* = 2), or not reporting tobacco use status during COVID (*n* = 3). This resulted in 291 (291/3396; 8.6%) participants for analysis. This research was approved by the Penn State College of Medicine Institutional Review Board (IRB).

### 2.2. Study Procedures

Upon clicking the unique survey link, participants were provided with a summary of the research. Participants provided implied consent by clicking to continue with the survey. All responses to the survey were anonymous. Participants did not receive an incentive for their participation. Participants were asked a series of questions about their experiences with COVID-19 including any symptoms that they experienced. Participants were asked, “Starting in March 2020, we all became aware of an infectious virus (Coronavirus or COVID-19) that has spread across the country and the world. At any point in time from February 2020 onwards, did you experience symptoms that made you believe you may have contracted the virus?” Participants reporting symptoms were asked to report all the symptoms that they experienced (check all that apply), whether they had been tested (yes/no), and what the test results were (positive/negative/pending). Participants were also asked to rate their perception of risk using two questions, “How do you perceive your risk of catching COVID-19 compared with those who do not use tobacco or nicotine products?” and “If you were to catch COVID-19, how do you perceive your risk of suffering serious complications compared with those who do not use tobacco or nicotine products?” Response options for both questions were much less likely, less likely, the same, more likely, and much more likely.

Participants were then asked a series of questions about their tobacco use. First, participants were asked to report on their tobacco use prior to the COVID-19 pandemic by asking, “Which of the following products did you use just prior to March 2020 (before the COVID-19 pandemic)?” Participants were instructed to select all products used from the following list: cigarettes, e-cigarettes/vape pens, cigars, pipes, snus/snuff/dip/chew, hookah/waterpipe, or dissolvables. Then, participants were asked to report on their tobacco use at the time of the survey (during the COVID-19 pandemic) by asking, “Do you currently use cigarettes, electronic cigarettes/vape pens, cigars, pipes, snus/snuff/dip, chew, hookah/waterpipe, or dissolvables?”. If participants endorsed a product, they were asked how many times per day they used the product. For e-cigarettes, a single “use time” was defined as approximately 15 puffs or 10 min of use. We asked participants the type of e-cigarette device currently used, from cigalike, advanced, mod, and pod-mod devices [25]. Additionally, participants were asked, “Did you change devices in response to the COVID-19 pandemic?” (yes/no). Those who responded yes were asked to describe the change (What device did you use? What do you use now? Why did you switch?).

Changes in use were evaluated by comparing product use times (per day) before and during the pandemic. Reasons for increases and decreases in use were assessed with two separate multiple choice questions (quantitative data). In addition, participants were asked an open-ended question to gain qualitative feedback about how COVID-19 has affected their tobacco use, “How has the COVID-19 pandemic affected your tobacco or nicotine products use? Please describe.”

Participants were also asked a series of questions about quitting their tobacco/nicotine product use, including questions about the importance of quitting and their confidence in doing so. Participants were asked, “How important is it to you to stop tobacco or nicotine products use now?” and “How confident are you that you will succeed in stopping your tobacco or nicotine products use now?” Response options ranged from 1 (not at all) to 10 (extremely). Finally, participants were asked whether they had made any quit attempts during the COVID-19 pandemic and whether their quit attempt was driven by their desire to reduce their risk for COVID-19. Participants were asked, “Have you made or are you making an attempt to quit your tobacco or nicotine products use during the COVID-19 pandemic?” (yes/no), and if yes, “Was your quit attempt motivated by a desire to reduce your risks from the Coronavirus?” (yes/no). Those who attempted to quit were asked to report the methods used to quit. All survey questions and response choices can be accessed from the Survey Questionnaire Supplement (File S1).

### 2.3. Data Analysis

Means and frequencies were used to describe the sample demographics, COVID-19-related questions, and perceptions of COVID-19 risk. Linear regression models were used to predict perception of COVID contraction risk and COVID perception of complications risk. Independent variables included in these models were demographics (gender, age, race, ethnicity, education), COVID symptoms (yes/no), cigarette use (yes/no), e-cigarette use (yes/no), and importance and confidence to quit.

McNemar’s tests were used to evaluate changes in the proportion of users reporting cigarette and e-cigarette use (yes/no) from pre-COVID to during the COVID-19 pandemic. Paired t-tests were used to evaluate within-subject changes in cigarettes smoked per day and e-cigarette times per day pre- to during COVID-19. Frequencies described the proportion of users who increased and decreased their use and their reasons for changes in use. Finally, a logistic regression model was used to predict attempting to quit. Independent variables included demographics, COVID symptoms (yes/no), cigarette use (yes/no), e-cigarette use (yes/no), importance and confidence to quit, and perception of contraction and complications risk.

A figure was generated to describe overall changes in tobacco use from pre-COVID to during COVID. For this figure, participants were classified into groups based on the tobacco products that they reported using pre- and during COVID. Exclusive cigarette smokers were those who reported only use of cigarettes, while exclusive e-cigarette users were those who reported only use of e-cigarettes. Dual users were those who reported use of both cigarettes and e-cigarettes. Products other than cigarettes or e-cigarettes were classified in a category as other which included cigars, pipes, or chew.

Qualitative data from the open-ended question regarding how the pandemic has affect tobacco use were in the form of brief comments. Comments were grouped together into general themes and sub-themes (if necessary). The themes derived from the open-ended question and example quotes are provided below.

## 3. Results

Participants (*n* = 291) were 25.6% male, 93.4% white, and 21.7% earned a college degree or greater. Participants had a mean age of 47.3 (SD = 11.6) years and smoked for an average of 27.5 years (SD = 12.1) (*n* = 267) at the time of survey. Nearly all participants were from Pennsylvania (98.9%). Overall, 21.7% of participants (*n* = 63) reported symptoms that they believed to be caused from COVID-19 starting from February 2020 onward. Among those who reported symptoms (*n* = 63), the most commonly reported symptoms were headache (66.7%), dry cough (66.7%), body aches (60.3%), and shortness of breath (49.2%). A total of 36.5% reported mild symptoms, 60.3% reported moderate symptoms, and 3.2% (*n* = 2) reported severe symptoms requiring hospitalization. Of the 14 participants (22.2% of those reporting symptoms) tested for COVID, 12 were negative (85.7%), and 2 were still pending (14.3%). The participants who reported severe symptoms requiring hospitalization did not report a test result. At the time of the survey, COVID-19 tests were not widely available, were generally for those experiencing symptoms or with known exposure, and the wait time for results was variable.

The majority of participants reported that they believed that their risk of contracting COVID-19 was the same as non-tobacco users (67.3%), while 6.5% believed that their risk was lower and 26.2% believed that their risk was higher. Perceiving higher risk was associated with being female (β = −0.29, *p* < 0.01) and reporting greater importance for quitting (β = 0.041, *p* < 0.01). A total of 57.7% believed that they were more at risk to suffer serious complications from COVID-19 if contracted, while 38.2% believed that their risk was the same, and 4.1% believed that their risk was lower. Perceiving higher risk for serious complications was associated with greater age (β = 0.01, *p* = 0.01), being female (β = −0.32, *p* < 0.01), reporting greater importance for quitting (β = 0.09, *p* < 0.01), and reporting lower confidence to quit (β = −0.05, *p* = 0.03),

Pre-COVID, 93.1% (*n* = 271) of participants reported current use of cigarettes. During COVID, there was a significant decrease in the number of cigarette users (90.4%) (*n* = 263) (*p* < 0.01) (Table 1). There was an overall increase in the number of cigarettes smoked per day (CPD) among those who continued smoking pre- and during COVID-19 (Table 1). Among only the smokers who increased (28.2%; *n* = 71), the number of cigarettes smoked per day rose from 13.3 (SD = 6.7) to 18.9 (SD = 8.9) CPD (*p* < 0.01). The top reasons for increasing use (quantitative data), among those who reported (*n* = 61), were stress (82.0%), more time to smoke (52.5%), and boredom (11.5%). A total of 11.1% (*n* = 28) decreased their use, from 18.8 (SD = 7.2) CPD to 9.8 (SD = 5.5) CPD (*p* < 0.01). The top reasons for decreasing use (quantitative data), among those who reported (*n* = 19), were health reasons (21.1%), schedule changes (31.6%) and being around other non-smokers (i.e., children) (31.6%). A total of 60.7% (*n* = 153) had no change in CPD.

There were 256 participants who gave qualitative responses on how the pandemic has changed their tobacco or nicotine product use. Themes derived from the qualitative responses are presented in Table 2.

**Figure 1:**
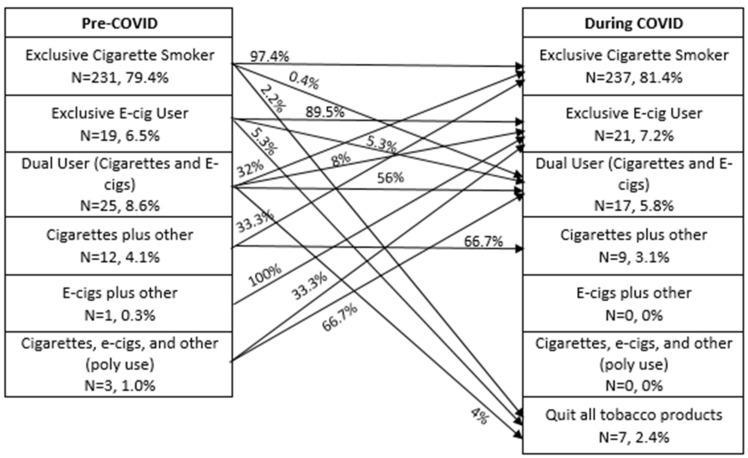
Pre-COVID-19 and during COVID-19 tobacco and nicotine use transitions.

There was also a decrease in the number of participants who reported e-cigarette use pre-COVID to during COVID (16.2%, *n* = 47 to 13.1%, *n* = 38, *p* < 0.01). Among the continued users, there was a significant increase in times per day from 13.1 (SD = 14.8) (*n* = 32), to 16.4 (SD = 16.3) (*n* = 37) (*p* = 0.01). The largest proportion of participants reported use of a pod-mod device (48.7%, *n* = 18), followed by mod devices (35.1%, *n* = 13), advanced (8.1%, *n* = 3), and cigalike devices (8.1%, *n* = 3). Of those who reported use of a pod-mod (*n* = 18), more than half reported use of a JUUL (55.6%, *n* = 10). Only one participant stated that they changed devices in response to the pandemic. However, the participant did not provide additional detail about what product they used prior to the pandemic or why they switched. Figure 1 displays the number and proportion of participants using each tobacco type and the proportion that transitioned in each category pre- and during COVID.

During the COVID-19 pandemic, participants reported that quitting was somewhat important (mean score 5.7) (SD = 3.0) (*n* = 275), but participants were not very confident in their ability to quit (mean score 2.8) (SD = 2.3) (*n* = 279). Approximately one-quarter of participants (24.5%) (*n* = 71) reported making an attempt to quit during the pandemic, with approximately one-third (33.8%) (*n* = 24) of those who attempted to quit reporting that their reason for quitting was to reduce their risk from COVID-19. Those who experienced symptoms associated with COVID were not more likely to attempt to quit (*p* = 0.24). Variables significantly associated with making a quit attempt were greater importance (β = 0.385, *p* < 0.01) and greater confidence (β = 0.161, *p* < 0.01) for quitting. Among those who made an attempt to quit (*n* = 71), the most commonly reported methods used to quit were cold turkey (28.2%), use of medications such as Chantix or Wellbutrin (26.8%), and use of nicotine-replacement therapies (NRT) (22.5%). Of those who attempted to quit, seven (9.9%) participants were successful in quitting all tobacco use (reported no tobacco use at the time of the survey), with three quitting cold turkey, two using NRT medications, and two using other medications. 

## 4. Discussion

The findings of this study provide preliminary insight into tobacco users’ perceived threat of COVID-19 infection and how these beliefs influenced their tobacco use behaviors during the early stages of the COVID-19 pandemic. We found that many tobacco users have increased their tobacco use (both cigarette and e-cigarette use) in response to the COVID-19 pandemic. Increases in tobacco use have been reported across the globe [26,27,28]. This is concerning because preliminary evidence suggests that tobacco use leads to greater susceptibility to complications from COVID-19 [8,9]. In addition, greater use could lead to greater dependence [29], which may prolong use and make quitting more difficult. The most commonly reported reasons for increased use were stress, more time available to use tobacco (e.g., working from home), and boredom while quarantined. Other reports have found similar reasoning for increased cigarette [30] and e-cigarette use during the pandemic [31]. While many increased their tobacco use, others indicated attempting to quit in response to the pandemic, with seven (7/291; 2.4%) achieving cessation. The majority of those who tried to quit reported doing so without using any FDA-approved medications. Quit success may be improved if more smokers use medications proven to increase the chance of achieving long-term abstinence [32]. It is known that many adults avoided medical care during the pandemic, even routine care which offers opportunities for discussions on tobacco use cessation [33]. Access to remote proven options for cessation such as home delivery of medications or tele-health counseling [34], as is being used in many other areas of medicine, would be well suited for tobacco treatment during the pandemic. In addition, as people are avoiding public settings, it should be considered how this affects social support systems such as group-based counseling. 

The majority of participants reported that they believed their risk of contracting COVID-19 was the same as that for non-tobacco users and 58% believed they were at a higher risk for suffering from serious complications compared to non-tobacco users. Perceptions of greater risks of both becoming infected and suffering severe complications were associated with an increased importance of quitting which suggests that smokers may be motivated to quit due to the threat of COVID-19. Smokers enrolled in smoking cessations trials during the pandemic reported that their motivation to quit was altered by the pandemic [35]. A study among smokers from the United Kingdom and Australia found that 45% of respondents wanted more information on smoking and COVID-19 health risks [36]. Health-based messaging on smoking and COVID-19 risk may be important during this time to capitalize on any increased interest in quitting among the population. Some messaging based on COVID-19 and quitting has been implemented [37]. The American Lung Association has provided guidelines for tele-health in the context of tobacco cessation [38].

The association of greater COVID-19 risk perceptions with greater importance in quitting fits into the theoretical framework of the Health Belief Model (HBM) [39,40]. The HBM is framed on a number of key health beliefs, such as perceived susceptibility, perceived severity, perceived benefits, perceived barriers, and self-efficacy that persuade individuals to make healthy behavior changes. The HBM has been used in previous studies to characterize response to health threats such as the swine flu, tuberculosis, and others [41,42,43,44]. Based on the framework, smokers who perceive themselves susceptible to a threat are more likely to engage in reduction or quitting behaviors. One-third of those who reported making a quit attempt during the pandemic in this study reported doing so to reduce their risk of COVID-19. However, many participants reported low confidence, or self-efficacy, in being able to quit, which highlights the importance of ongoing tobacco cessation messaging during this time. Preventative health interventions and messaging based on the HBM have been used in the past to increase a variety of healthy behaviors [45,46,47,48]. Cessation messaging and interventions within the context of the pandemic that are grounded in the HBM would be good opportunities for future research in order to increase self-efficacy and motivation to quit tobacco use at this time.

The following limitations should be taken into consideration. The sample of tobacco users in this study were recruited from a tobacco user registry of individuals who are interested in participating in tobacco-related studies, so the sample is not nationally representative. However, our findings regarding patterns of data on use and quitting behaviors are consistent with those from two other recent studies around the same time period [22,24]. The low response rate could have been due to the lack of incentive for completing the survey. As this study was conducted during the pandemic when research activities were limited, biochemical validation of nicotine exposure and quitting was not performed. All responses were self-reported and may be subject to recall bias due to retrospective reporting. Causality cannot be determined regarding some of the identified associations given the survey was cross-sectional. Lastly, the data presented within this paper were collected at the beginning of the pandemic in the U.S. when lockdowns were being implemented and less information was known about the virus. Pennsylvania, like many other states in the surrounding area, was under strict social distancing rules with stay-at-home orders in place, mask mandates, and non-essential businesses and schools closed which directly influenced some of the responses we collected.

In conclusion, this study found that tobacco use increased among a sample of existing users during the COVID-19 pandemic. These results may have several implications for public health. Increased use is associated with higher dependence, exposure to tobacco-related toxicants, and increased financial burden. Tobacco cessation efforts should not become underemphasized because of the pandemic. There is a need for innovative methods to support users who are interested in quitting during this particularly difficult time. The fact that many smokers are aware of their increased health risks of serious complications with COVID-19, are interested in quitting, and are spending more time at home suggests that smokers may be particularly receptive to pro-active telephone smoking cessation counseling during the pandemic.

## Figures and Tables

**Table 1:** Pre- and current tobacco use during the COVID-19 pandemic.

	Pre- COVID-19	During COVID-19	*p* Value
Cigarette smoker % (n)	93.1 (271/291)	90.4 (263/291)	<0.01
Cigarettes per day Mean (SD) (n)	15.4 (7.7) (258)	16.3 (8.4) (253)	<0.01
E-cigarette user % (n)	16.2 (47/291)	13.1 (38/291)	<0.01
Times used per day Mean (SD) (n)	13.1 (14.8) (32)	16.4 (16.3) (37)	0.01

**Table 2:** Qualitative themes and responses regarding COVID-19-related tobacco use changes ^1^.

Qualitative Response Themes	Sub-Themes	Response Example
Increase smoking		
	Boredom	*“I smoke more than I used [to] because I’m stuck in the house and because I feel bored”* *“Since I’m stuck in the house, I have smoked more...probably out of boredom. I hate it.”*
	Stress/anxiety	*“The stress of going to work and coming home and possibly affecting my family. Every time going to the grocery store to get food and having to come home and wash everything so there is no chance of catching it. Stress of not being able to pay my bills on time or at all.”*
	Working from home	*“I smoke more, as I am working from home. I can easily walk outside in between work functions. And with trying to be a teacher, mom, employee and everything else- it can make for a stressful day.”* *“Working at home allows me to smoke at will rather than being in a smoke free environment for 8 hours per day.”*
Decreased smoking		
	Health reasons	*“I am trying not to smoke as much due to the fact of my hands and fingers being in close contact with my face, especially mouth.”* *“It has caused me to cut back, just in case I would contract the virus.”*
	More time at home	*“Smoke less because I am home more.”* *“Being stuck in the house with my girlfriend has resulted in her taking notice to my habit more often. That has then affected how many times she says “you smoke too much” in a day. For the record, its increased. THAT in return has reduced the amount of times I go outside for a cigarette.”*
No change		*“The COVID-19 pandemic has not affected my tobacco products use at all. I still purchase, while wearing a mask.”* *“It has not affected the number of cigarettes I smoke each day; however, since this coronavirus pandemic, I tend to feel as though I might be being judged (by strangers passing by) more than usual.”*
Desire to quit/reduce		*“I talked to my physician about quitting smoking and got the nicotine patch from pharmacy. I haven’t used it yet. I smoke when I’m stressed and anxious so I don’t want to go through even more stress, anxiety from withdrawal at this time.”* *“I quit as soon as I came down with a fever and cough. Clearly I am aware of how detrimental smoking is to my health; however, I did not consider how it could make me more vulnerable to COVID-19 and its effects. I was terrified and quit immediately.”* *“I was in the process of quitting and this scare was enough to make me quit. Although as this drags on I find myself having the urge to smoke a cigarette again.”*
Changes in obtaining nicotine products		*“It has made it more difficult to get as this is a non- essential business. Luckily though they were able to go online.”* *“I just buy them in bulk now”*

^1^ 35 participants did not provide a response.

## Data Availability

The data presented in this study are available on request from the corresponding author.

## Supplementary Materials

The following are available online at https://www.mdpi.com/1660-4601/18/4/1795/s1, File S1: COVID-19 Questions.
