# Supplementary material for: Tobacco Use Changes and Perceived Health Risks among Current Tobacco Users during the COVID-19 Pandemic
Source: Int J Environ Res Public Health. 2021 Feb 12;18(4):1795. doi: 10.3390/ijerph18041795 (PMC7917755; doi:10.3390/ijerph18041795)
Supplement: Supplementary file 1 [file ijerph-18-01795-s001.pdf]

COVID-19 Questions:

Starting in March 2020, we all became aware of an infectious virus (Coronavirus or COVID-19) that has spread across the country and the world. At any point in time from February 2020 onwards, did you experience symptoms that made you believe you may have contracted the virus?

☐ 1, Yes

☐ 0, No

From the symptoms listed below, please check the ones you experienced during a single 14-day period since February 2020:

Dry cough
High temperature/fever
Shortness of breath
Tiredness/fatigue
Loss of taste
Loss of smell
Stomach pain
Chest pain
Diarrhea
Watery eyes
Itchy eyes
Runny nose
Stuffy nose
Sneezing
Body aches
Headaches
Sore throat
Vomiting
Other

How severe would you rate these symptoms:

Mild
Moderate
Severe (Required hospitalization)

Were you told by a medical person that you likely contracted COVID-19?

☐ 1, Yes

☐ 0, No

Have you ever tried to get tested for COVID-19?

☐ 1, Yes

☐ 0, No

If yes, what were the test results?

☐ 1, Test is still pending

☐ 2, Positive (COVID-19 Virus detected)

☐ 3, Negative (COVID-19 Virus not-detected)

☐ 4, Was not able to get tested

If no, would you like to be tested for having COVID-19?
- [ ] 1, Yes
- [ ] 0, No

Have you been in close contact with someone who was diagnosed with COVID-19?
- [ ] 1, Yes
- [ ] 0, No

If yes, briefly summarize the situation and nature of your contact: ____________

If there was a test (typically called an "antibody test") that could tell you whether you had already been exposed to Coronavirus, and are therefore very unlikely to get COVID-19 illness in future, would you want to take that test?
1. No
2. Yes probably (depends on cost, etc)
3. Yes definitely
4. Unsure

Has a doctor or medical provider ever told you that you have any of the following? Please check all that apply:
1. Heart disease
2. COPD/Emphysema
3. Asthma
4. Diabetes
5. Depression/Anxiety
6. Immunocompromised (or have a weakened immune system)
7. Kidney Disease
8. Liver Disease
9. Cancer (Current)
10. Cancer (In remission)
11. High Blood Pressure/Hypertension

What is the main impact that Coronavirus has had on you personally? (could be health, employment, lifestyle, relationships, etc?) Please describe and be as specific as possible.

____

Has your employment situation changed in response to COVID-19?
1. No, my employment situation is the same
2. Yes, Currently unemployed (due to COVID-19)
3. Yes, Currently working from home (due to COVID-19)
4. Yes, Currently working more hours (due to COVID-19)

Yes, Currently employed but working less or zero hours (due to COVID-19)**Which of the following activities have you been consistently practicing to reduce your risks of contracting COVID-19? Select all that apply.**
1. Keeping at least 6 feet away from people other than those I live with, at all times
2. Staying at home other than occasionally leaving when absolutely necessary (get food, walk a pet, go to work, get some fresh air/exercise)
3. washing my hands after every time I may have touched something that could have been contacted by the virus

4. wearing a face mask when outside in public or potentially near other people
5. wiping down my itemsand surfaces with disinfectant when I come back from shopping.
6. keeping my shoes outside so I don't risk bringing coronavirus into the house
7. reduced my tobacco consumption as it may affect my risks from coronavirus
8. working from home so as to avoid contact with people at work

Which of these has been the most difficult to maintain? Select one.

1. Keeping at least 6 feet away from people other than those I live with, at all times
2. Staying at home other than occasionally leaving when absolutely necessary (get food, walk a pet, go to work, get some fresh air/exercise)
3. washing my hands after every time I may have touched something that could have been contacted by the virus
4. wearing a face mask when outside in public or potentially near other people
5. wiping down my items and surfaces with disinfectant when I come back from shopping.
6. keeping my shoes outside so I don't risk bringing coronavirus into the house
7. reduced my tobacco consumption as it may affect my risks from coronavirus
8. working from home so as to avoid contact with people at work

How much longer do you think you and your family could continue to practice the social distancing and other activities that we have all been told to practice?

1. A week or two
2. 3-4 weeks
3. 5-6 weeks
4. 7-8 weeks
5. 3-5 months
6. 6 months or more

How do you think public health professionals and others could do a better job of managing the COVID-19 pandemic better in this country, in your opinion? Please describe and be as specific as possible.

____

Tobacco Use Questions:

Have you <u>ever</u> regularly used cigarettes, electronic cigarettes/vape pens, cigars, pipes, snus/snuff/dip, chew, hookah/waterpipe, or dissolvables?

☐ 1, Yes
☐ 0, No

Where you regularly using cigarettes, electronic cigarettes/vape pens, cigars, pipes, snus/snuff/dip, chew, hookah/waterpipe, or dissolvables just prior to March 2020 (before the COVID-19 pandemic)?

☐ 1, Yes
☐ 0, No

Which of the following products did you use just prior to March 2020 (before the COVID-19 pandemic)?

☐ 1, Cigarettes
☐ 2, Pipes
☐ 3, Snus/Snuff/Dip

☐ 4, Chew
☐ 5, Electronic nicotine product (e-cigs, vape pens, hookah pens, vaporizers)
☐ 6, Hookah/water pipe
☐ 7, Dissolvable tobacco (lozenge, strips, or sticks)
☐ 8, Cigars

[1] How many cigarettes per day did you smoke at that time? ____
[2] How many pipes per day did you smoke at that time? ____
[3] How many times per day did you use snus/snuff/dip at that time? ____
[4] How many times per day did you use chew at that time? ____
[5] How many times per day did you use your electronic cigarette at that time? [assume that one "time" consists of around 15 puffs or lasts around 10 minutes]
[6] How many times per day did you use hookah/waterpipe at that time? ____
[7] How many times per day did you use dissolvable tobacco at that time? ___
[8] How many cigars per week did you smoke at that time? _____

During that time, how strong were your urges to use tobacco or nicotine products?

1. None
2. Slight
3. Moderate
4. Strong
5. Very Strong
6. Extremely Strong

Do you currently use cigarettes, electronic cigarettes/vape pens, cigars, pipes, snus/snuff/dip, chew, hookah/waterpipe, or dissolvables?

1, Yes
0, No

Which of the following products do you currently use?

☐ 1, Cigarettes
☐ 2, Pipes
☐ 3, Snus/Snuff/Dip
☐ 4, Chew
☐ 5, Electronic nicotine product (e-cigs, vape pens, hookah pens, vaporizers)
☐ 6, Hookah/water pipe
☐ 7, Dissolvable tobacco (lozenge, strips, or sticks)
☐ 8, Cigars

[1] How many cigarettes per day do you smoke? ____
[2] How many pipes per day do you smoke? ____
[3] How many times per day do you use snus/snuff/dip? ____
[4] How many times per day do you use chew? ____
[5] How many times per day do you use your electronic cigarette? [assume that one "time" consists of around 15 puffs or lasts around 10 minutes]

[6] How many times per day do you use hookah/waterpipe? ____
[7] How many times per day do you use dissolvable tobacco? ___
[8] How many cigars per week do you smoke? _____

In the past 7 days, how strong were your urges to use tobacco or nicotine products?

1. None
2. Slight
3. Moderate
4. Strong
5. Very Strong
6. Extremely Strong

[for each product used – either prior to March 2020 or now]
When was the last time you used [product name]?

☐ 0, Today
☐ 1, Days→ how many days ago?
☐ 2, Months→ how many months ago
☐ 3, Years → how many years ago

[If reported cigarette use– either prior to March 2020 or now:]
How many years total have you smoked? _________________

[If reported e-cig use – reported currently:]
What type of e-cig do you use most often? Please select the type that most closely resembles your device.

Type 1 –<br>Cigalike device<br>- Battery similar in size and shape to cigarette with e-liquid housed in cartridges<br>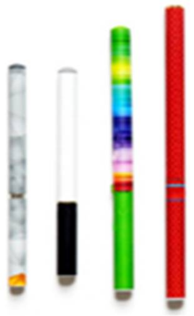	Type 2 –<br>Advanced Device<br>- Pen-shaped battery with e-liquid housed in tanks<br>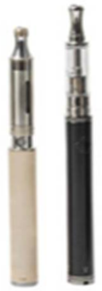	Type 3 – Mod Device<br>- Large battery modified from standard cigarette shape with e-liquid housed in tanks<br>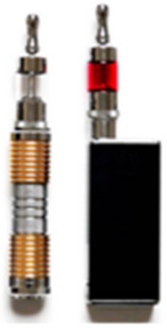	Type 4 – Pod Mod Device<br>- Small, sleek battery with e-liquid housed in pods<br>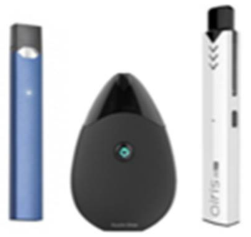

What is the brand name of the e-cig you use most often?

1, Aspire
2, Blu

3, eGo
4, Eleaf
5, GeekVape
6, Green Smoke
7, Halo
8, iStick
9, Joyetech
10, Juul
11, KangerTech
12, Logic
14, Mig Vapor
15, NJOY
16, Om Vapors
17, SMOK
18, South Beach
19, V2
20, Vapor4Life
21, VaporFi
22, Vuse
23, Don't know
888, Other
If other, brand name:

Did you change devices in response to the COVID-19 pandemic?
1. Yes
2. No

If yes, please describe in detail. (What device did you use? What do you use now? Why did you switch?)
____

How has the COVID-19 pandemic affected your tobacco or nicotine products use. Please describe and be as specific as possible.

____
____

[If reported use increased:]
Why is your use increasing? Check all that apply.

1. More time available to use tobacco/nicotine products
2. Stress
3. Other: Please Explain

[If reported use decreased:]
Why is your use decreasing? Check all that apply.

1. Money issues
2. Tobacco/nicotine products are hard to obtain
3. Health reasons
4. Schedule changes (ie. busier)

5. Around others more often (ie. kids are home)
6. Other: Please explain

Have you had any issues obtaining the tobacco or nicotine products that you wanted during the COVID-19 pandemic?

1. Yes
2. No

If yes, what issues did you experience: ____________________

If yes, what extra efforts did you have to make to obtain your tobacco or nicotine products? _____

How important is it to you to stop tobacco or nicotine product use now?

1 Not at all important 2 3 4 5 6 7 8 9 10 Extremely Important

How confident are you that you will succeed in stopping your tobacco or nicotine product use now?

1 Not at all confident 2 3 4 5 6 7 8 9 10 Extremely Confident

Have you made or are you making an attempt to quit your tobacco or nicotine product use during the COVID-19 pandemic?

1. Yes
2. No

If yes, what method did you use?

1. Cold turkey/just stopped
2. Used nicotine replacement therapy (NRT) like the patch, gum, or lozenge
3. Used medications like Chantix or Wellbutrin
4. Telephone Quitline
5. Internet site (e.g. Smokefree.gov or BecomeanEx.com)
6. Other: _____________

If yes, was your quit attempt motivated by a desire to reduce your risks from the Corona virus?

1. Yes
2. No

How do you perceive your risk of catching COVID-19 compared with those who do not use tobacco or nicotine products?

1. Much less likely
2. Less likely
3. The same
4. More likely

5. Much more likely

If you were to catch COVID-19, how do you perceive your risk of suffering serious complications compared with those who do not use tobacco or nicotine products?

1. Much less likely
2. Less likely
3. The same
4. More likely
5. Much more likely

What do you think public health professionals and others should know about how they could do a better job of helping people to reduce or quit tobacco or nicotine product use and improve their health, in your opinion? Please describe and be as specific as possible.

____

Demographic Questions:

What is your biological sex? [gender]

☐ 0, female
☐ 1, male
☐ 2, Other

Do you consider yourself Hispanic or Latino? [ethnicity]

☐ 1, Yes
☐ 0, No

What race best describes you? [race]

☐ American Indian or Alaska Native
☐ Asian
☐ Black or African American
☐ Native Hawaiian or Other Pacific Islander
☐ White
☐ Other
Other, please describe: __________

What is the *highest* level of school you have completed or the highest degree you have received? [education]

☐ 1, I did not complete high school and I do not have a GED
☐ 2, I graduated from high school or I received a GED
☐ 3, I have completed some college, technical school, or I have an Associate's Degree
☐ 4, I have graduated from a college or university with a Bachelor's degree or more

During this time of COVID-19, how often do you see or talk to people that that you care about and feel close to? (For example: talking to friends on the phone, visiting friends or family, going to church or club meetings)

1. Less than once a week
2. 1 or 2 times a week

3. 3 to 5 times a week
4. 5 or more times a week

Stress is when someone feels tense, nervous, anxious, or can't sleep at night because their mind is troubled. How stressed are you currently?

1. Not at all
2. A little bit
3. Somewhat
4. Quite a bit
5. Very much

Are you covered by health insurance or some other kind of health care plan?

1. Yes
2. No
